# The Classification and Surgical Treatments in Adult Hirschsprung’s Disease: A Retrospective Study

**DOI:** 10.3389/fmed.2022.870342

**Published:** 2022-04-08

**Authors:** Shengzhe Ma, Yue Yu, Anfu Pan, Haifeng Gong, Zheng Lou, Lianjie Liu, Liqiang Hao, Ronggui Meng, Jinke Sui, Wei Zhang

**Affiliations:** Department of Colorectal Surgery, Changhai Hospital, Shanghai, China

**Keywords:** Hirschsprung’s disease (HD), surgical methods, megacolon, pathogenisis, retrospective study

## Abstract

**Purpose:**

To explore the treatments and short-term effects of different types of adult Hirschsprung’s disease.

**Methods:**

89 patients treated in Shanghai Changhai Hospital were retrospectively analyzed. According to the patient’s medical history, clinical manifestations, auxiliary examination and postoperative pathological results, the patients were divided into adult congenital megacolon, adult idiopathic megacolon, ganglion cell deficiency (types I and II), toxic megacolon and iatrogenic megacolon, The Treatment methods and short-term prognosis of patients in each group were summarized.

**Results:**

41 cases of Hirschsprung’s disease in adults and low anterior resection or pull-out low anterior resection was performed, and 35 patients with idiopathic Megacolon were treated with one-stage subtotal colon resection under the condition of adequate preoperative preparation. Some patients admitted for emergency intestinal obstruction received conservative treatment first or underwent elective surgery after colonoscopic decompression was improved; two patients with ganglion cell deficiency subtotal colectomy were performed to remove the dilated proximal bowel segment and the narrow distal bowel segment; three patients with toxic Hirschsprung’s disease underwent colostomy in mild cases, while subtotal colorectal resection was required in severe cases; Iatrogenic megacolon was diagnosed in eight cases and the optimum operation should be selected according to the specific conditions of patients.

**Conclusion:**

Adult Hirschsprung’s diseases were divided into adult congenital hirschsprung’s disease, idiopathic Hirschsprung’s disease, ganglion cell deficiency, toxic hirschsprung’s disease, and iatrogenic Hirschsprung’s disease. Different types of surgical treatments for Hirschsprung’s disease in adults should be selected according to the specific diagnosis. All patients with adult Hirschsprung’s diseases have good short-term outcomes after surgical treatment.

## Introduction

Adult Hirschsprung’s disease (HD) is a relatively rare type of disease (1, 2). Since first report by Professor Hirschsprung in 1886, the treatment of adult HD has been explored for more than 200 years (3). However, due to the lack of understanding of its pathogenesis, many patients are misdiagnosed as constipation with attempted treatment with laxatives, irrigation, or manual disimpaction (4). With the deepening understanding of the disease, the pathogenesis and classification have been clarified. Among which, the most common one is adult congenital megacolon, followed by adult idiopathic megacolon, and relatively rare ones of ganglion cell deficiency (type I and II), toxic megacolon, and iatrogenic megacolon (5). As one of the earliest centers to carry out adult HD surgery in China, our department has been studying adult HD since the 1970s and has accumulated experience of the treatment of different types of HD. This study conducted a retrospective analysis of 89 cases of HD admitted and treated in our department. We summarized the clinical characteristics and surgical procedures of different types of adult HD, and discussed the surgical options for adult HD.

## Materials and Methods

### Data Collection

Eighty-nine patients admitted to the Department of Colorectal Surgery, Changhai Hospital between April 2000 to April 2020 were included in this study. Those included patients were diagnosed with adult HD by CT, barium enema, defecography, rectal suppression reflex, and pathological examination. Using a standardized data collection template, variables including patient demographics, type of HD, and complications were collected. Descriptive analyses were performed, and the results are presented as the mean ± standard deviation (SD).

### Surgical Technique

For patients diagnosed with adult congenital megacolon, the stenosis, transitional and obvious expansion parts were surgically removed, and the expanded and thickened proximal intestine were also resected. Multiple full-thickness frozen biopsies of the diseased rectum were conducted during operation to guide the resection margins. Specifically, the disassociation was conducted along the proper fascia of rectum down to the level of the levator ani muscle, and the mesorectum was isolated at the end of the rectum and the rectum were disconnected using Endo-GIA stapler. The expanded and thickened intestine was removed to the normal intestine at the proximal end, and the proximal end of colon and rectum were anastomosed end-to-end using tubular anastomat. If tension existed at the anastomosis site, the splenic flexure of colon would be freed. Moreover, preventive terminal ileostomy can be conducted if the anastomosis was at a low position. A negative pressure drainage tube was placed in the anterior sacrum and will be removed 1 week after the operation. The stoma will be returned 3–6 months postoperatively after evaluating the conditions of patients.

For patients diagnosed with adult idiopathic megacolon, Hirschsprung’s colon resection can be conducted. Specifically, this procedure involved subtotal colon resection with ileal rectal anastomosis or ascending colorectal anastomosis. For patients admitted with emergency intestinal obstruction, conservative treatment or selective surgery after colonoscopic decompression was conducted. If conservative treatment was ineffective, emergency stoma surgery will be performed. With adequate preoperative preparation, the second-stage surgical treatment can be conducted, which was subtotal colon resection with ileorectal anastomosis or ascending colorectal anastomosis, and the stoma was removed at the same time.

For patients with ganglion cell deficiency, subtotal colectomy can be chosen, which removes both the dilated proximal intestine and the narrow distal intestine. Since the rectum was less affected in this type of HD, ileorectal anastomosis or ascending colorectal anastomosis can be performed. For toxic megacolon, colostomy can be chosen in mild patients, and subtotal colon resection was performed in severely infected patients. In iatrogenic megacolon, Surgically removing iatrogenically induced stenosis intestine and the proximal part of expanded intestine and anastomosing the proximal colon with the rectum was a choice for those patients.

### Statistical Analysis

Categorical variables were described as frequency and percentage, and continuous variables were expressed as mean ± SD. Patients’ demographic and clinical characteristics were compared using the Pearson’s chi-squared test for categorical variables, and the student’s *t*-test for continuous variables. The statistical analysis was carried out using the SAS 9.4 software (SAS Institute Inc., Cary, NC, United States). *P* < 0.05 was considered statistically significant.

## Results

From April 2000 to April 2020, 89 patients diagnosed with adult HD were admitted to the Department Colorectal Surgery of Changhai Hospital and received surgical treatment. 49 males and 40 females aged 16 to 73 were included, with an average of 43.61 years. Informed consent for surgery was obtained and signed by all the patients. Among them, 41 cases were diagnosed with congenital megacolon, 35 cases with adult idiopathic megacolon, two cases with ganglion cell deficiency, three cases with toxic microcolon and eight cases of iatrogenic megacolon.

Among them, 48 patients had different degrees of abdominal distension, 33 had abdominal pain. All 89 patients had no history of mental illness, while 14 patients had systemic diseases, of which two patients were diagnosed with diabetes, 11 with hypertension, and one with both hypertension and diabetes. Five patients were diagnosed with organic gastrointestinal disease, which included four cases of diverticula and one case of gastrointestinal tumor. Among all the patients, 14 were admitted to the emergency department due to acute intestinal obstruction. The American Society of Anesthesiologists physical status (ASA-PS) was performed before operation, and 30 cases were evaluated as class I, 50 cases as class II, and 9 cases as class III (Table 1).

**TABLE 1:** Patient characteristics.

Characteristics	Quantity (%)
**Age, yr**	
Mean ± SD	43.61 ± 15.782
Range	16–73
**Sex**	
Female	40
Male	49
**Classification**	
Congenital megacolon	41 (46.1%)
Adult idiopathic megacolon	35 (39.3%)
Ganglion cell deficiency	2 (2.25%)
Toxic megacolon	3 (3.38%)
Iatrogenic megacolon	8 (7.87%)
**Mental illness**	
With	89 (100%)
Without	0 (0%)
**Systemic diseases**	
None	75 (84.3%)
Diabetes	2 (2.2%)
Hypertension	11 (12.4%)
Hypertension + diabetes	1 (1.1%)
**Organic gastrointestinal disease**	
None	84 (94.4%)
Tumor	1 (1.1%)
Diverticula	4 (4.5%)
**Digestive motility disorder**	
Without	89 (100%)
With	0 (0%)
**Initial symptom**	
Abdominal distension	69 (77.5%)
Abdominal pain	16 (18.0%)
Diarrhea	4 (4.5%)
**ASA**	
Class I	30 (33.7%)
Class II	50 (56.2%)
Class III	9 (10.1%)
**Emergency admission**	14 (15.7%)

### Surgical Treatment of Adult Hirschsprung’s Disease

#### Congenital Megacolon

**FIGURE 1:**
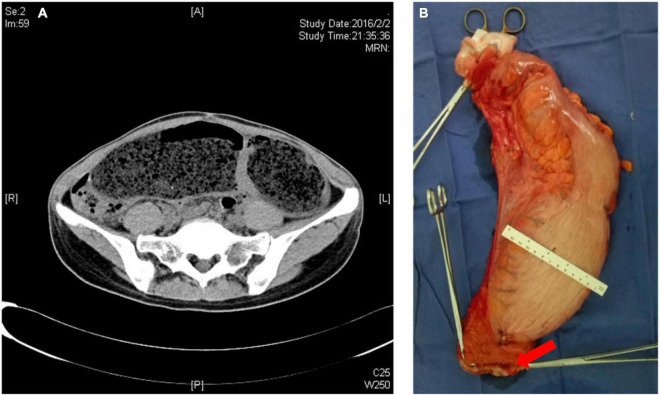
**(A)** CT image of adult congenital megacolon; **(B)** resected specimen of adult congenital megacolon (red arrow: the stump of the rectum).

Among the 41 patients with congenital megacolon, 23 underwent low anterior rectal resection and terminal ileostomy, six underwent pull-out anterior rectal resection and terminal ileostomy, and 12 underwent low anterior rectal resection. Moreover, prophylactic terminal ileostomy was performed in 29 patients due to the low position of anastomosis (Figure 1).

#### Adult Idiopathic Megacolon

**FIGURE 2:**
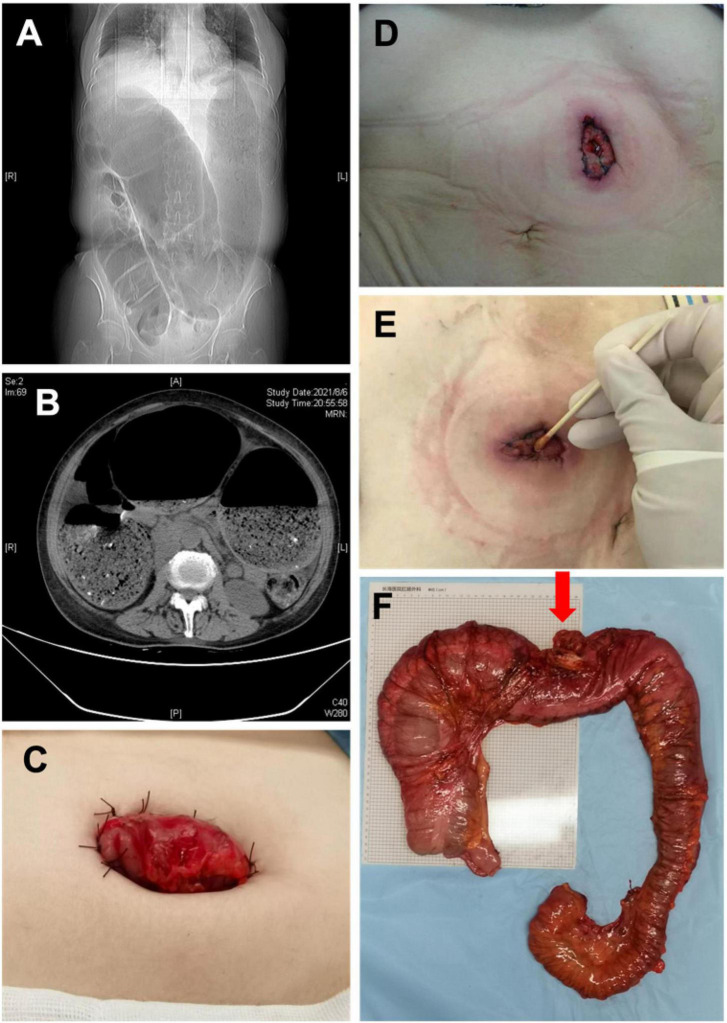
**(A)** X-ray image manifestations of adult idiopathic megacolon; **(B)** CT image of adult idiopathic megacolon; **(C)** photos after colostomy; **(D)** 1 week after colostomy; **(E)** 1 month after colostomy; **(F)** the intestine specimen after subtotal resection (Red arrow: transverse colostomy).

Thirty-five patients were diagnosed with idiopathic megacolon before surgery. Selective surgical treatment was conducted in 24 patients, which was subtotal colon resection with ileal rectal anastomosis or ascending colorectal anastomosis. Eleven cases were admitted to hospital with emergency bowel obstruction, accounting for 31.4% of patients with idiopathic megacolon. Among them, five cases were treated conservatively or selective surgery after colonoscopic decompression. The other six cases were treated by ostomy surgery. The loop colostomy cannot be performed in those six patients due to the ostomy bowel segment was extremely dilated, a transverse colon fenestration colostomy was performed. Unfortunately, various degrees of stoma stenosis occurred after the operation in those patients, only a cotton swab can be passed through the stoma in severe cases. All six patients with stoma underwent second-stage surgery, which was subtotal colon resection with ileorectal anastomosis or ascending colorectal anastomosis, and the stoma was removed at the same time (Figure 2).

#### Ganglion Cell Deficiency

**FIGURE 3:**
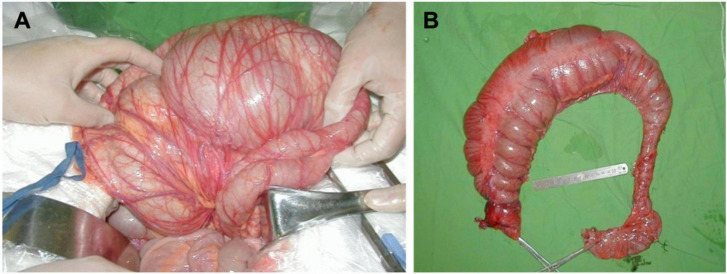
**(A)** Intraoperative photo of ganglion cell deficiency; **(B)** specimen of ganglion cell deficiency.

Since the clinical features of ganglion cell deficiency are indistinguishable from congenital megacolon, two patients were both diagnosed as congenital megacolon before operation. The diagnosis was corrected as ganglion cell deficiency based on the postoperative pathological results. The narrowed segment was mainly located in the descending colon or sigmoid colon. The number of ganglions in the diseased intestinal segment was significantly lower, and the proximal intestinal segment was severely expanded with megacolon-like changes (Figure 3). The surgical method was the same as that of idiopathic megacolon, which was removing both the dilated proximal bowel segment and the narrow distal bowel segment. The rectum was less involved in this type of patients, so an ileorectal anastomosis or ascending colorectal anastomosis was performed.

#### Toxic Megacolon

The three include patients had severe ulcerative colitis with fever and abdominal distension. CT showed the colon had megacolon-like changes with intestinal wall edema. Exhaust and defecation were not impeded, and mucus or necrotic fragments of intestinal mucosal tissue may appear in feces. One of the patients was accompanied by septic shock. In those cases, the intestinal mucosal barrier was destroyed, and bacterial toxins entered the blood. Thus, serious consequences may occur if the infection cannot be effectively controlled. Two mild cases of the three patients underwent colostomy, and subtotal colectomy were performed in the other severely infected patient because colostomy alone could not effectively alleviate the infection. This patient healed after active treatment.

#### Iatrogenic Megacolon

**FIGURE 4:**
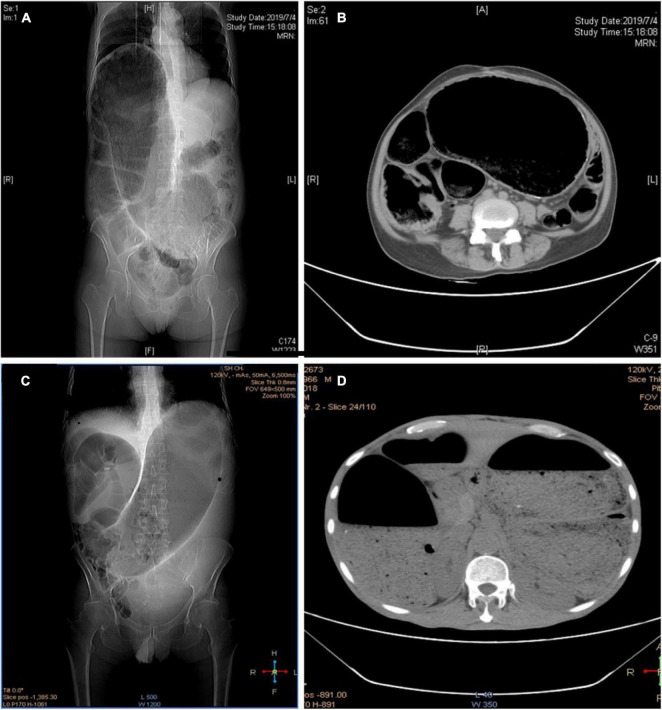
**(A,B)** CT image of iatrogenic megacolon caused by colonic anastomotic stenosis; **(C,D)** CT images of iatrogenic megacolon after radiotherapy for rectal cancer.

The local colon or a section of intestinal tube of the eight patients was narrow or lack of peristalsis because of various medical reasons, which eventually led to chronic expansion and the formation of megacolon. One of them was caused by anastomotic stenosis after descending colon cancer, who healed after the removal of the narrow anastomosis and the proximal part of megacolon. Another patient underwent colostomy due to chronic constipation, and the stoma was returned 2 years postoperatively. However, the anastomotic stenosis led to the formation of megacolon. He was admitted through emergency admission with obstructive symptoms. The proximal intestinal cavity was found extremely dilated and compressed the diaphragm and mediastinum. Bowel decompression and colostomy was performed because the condition did not improve after colonoscopic decompression (Figures 4A,B). In the other six patients, rectal wall stiffness or scar hyperplasia around the rectum caused by rectal cancer radiotherapy eventually led to chronic expansion of the proximal intestinal cavity and form megacolon. Those patients had repeated symptoms of abdominal distension, and two of them came to the hospital due to obstruction. The symptoms can be temporarily relived by conservative treatment (such as colonoscopy decompression, indwelling anal canal), but a permanent intestinal stoma was the best choice for them (Figures 4C,D).

### Postoperative Situation and Perioperative Safety

All patients recovered and discharged after the operation, and no death occurred. The median length of stay for all patients was 19.5 days. Complications occurred in 10 cases (11.24%), including postoperative intestinal obstruction (3 cases, 3.37%), severe hypoproteinemia (3 cases, 3.37%), postoperative anemia and hypovolemia (2 cases, s2.25%), abdominal effusion (1 case, 1.12%), and incision infection (1 case, 1.12%). Thirty-one patients underwent intraoperative or postoperative blood transfusion due to a large amount of postoperative exudation, anemia, or insufficient blood volume. One case of anastomotic leakage after operation was cured after conservative treatment (Table 2).

**TABLE 2:** Surgical method and complications.

Efficacy and complications	Quantity (%)
**Surgical method**	
Diversion	11 (12.4%)
Reconstruction	78 (87.6%)
**Intraoperative blood transfusion**	31 (34.8%)
**Classification of incision**	
I	0 (0%)
II	89 (100%)
III	0 (0%)
**Hospitalization, days (median, quartiles)**	19.5 (15,26)
**Complications**	
None	79 (83.9%)
Hypoproteinemia	3 (4.8%)
Anemia	2 (3.2%)
Abdominal effusion	1 (1.6%)
Recurrent obstruction	3 (4.8%)
Incision infection	1 (1.6%)

### Short-Term Surgical Results

The number of bowel movements in patients who did not undergo an intestinal stoma increased in the early postoperative period, and gradually decreased after drug treatment, which was less than 5 times a day after 3 months. Stoma was returned 3–6 months postoperatively for patients who underwent prophylactic terminal ileostomy and colostomy. The number of bowel movements also increased in the early postoperative period and can be reduced after drug treatment.

### Differences Among Treatment Modalities

Subsequently, we divided the patients into two groups according to the surgical methods, which is 11 patients who underwent fecal diversion (colostomy, etc.) and 78 intestinal continuity restoration (subtotal colectomy, small enterorectal anastomosis, etc.). Then, the clinical and pathological data, and the postoperative efficacy and complications within 30 days were analyzed (Table 3). Categorized by the surgical method, the preoperative gastrointestinal organic lesions (*p* = 0.026), the duration of obstruction (*p* = 0.024) and the incidence of postoperative complications (*p* = 0.031) were significantly higher in the fecal diversion group than that of the intestinal continuity restoration group.

**TABLE 3:** Differences among treatment methods.

	Diversion (11 cases)	Reconstruction (78 cases)	*P*
**Age, year, Mean ± SD**	54.4 ± 10.6	42.2 ± 15.9	0.053
**Sex**			
Male	6/11	43/78	
Female	5/11	35/78	0.897
**Hospitalization (median, quartiles), days**	17 (13, 26)	20 (15, 26)	0.834
**Systemic diseases**	3/11	11/78	0.342
**Organic gastrointestinal disease**	2/11	3/78	0.026
**Surgical complications**	3/11	7/78	0.031
**ASA**			
Class I	4/11	26/78	
Class II	4/11	46/78	
Class III	3/11	6/78	
**Intraoperative blood transfusion**	5/11	26/78	0.229
**Second operation**	2/11	5/78	0.125
**Emergency admission**	2/11	10/78	0.073
**Intraoperatve blood loss (median, quartiles), mL**	150 (100, 800)	200 (200, 400)	0.071
**Obstruction duration (median, quartiles), days**	25 (3, 45)	15 (9, 35)	0.024

## Discussion

Different types of adult HDs have different causes and varied treatment options. Therefore, the treatment of adult HD requires a definite diagnosis. Among them, adult congenital megacolon and adult idiopathic megacolon are relatively easy to differentiate and diagnose (6). However, ganglion cell deficiency is easily misdiagnosed as adult congenital megacolon (7, 8). A meta-analysis by Dingemann analyzed 11 articles about ganglion cell deficiency in 92 patients aged 4.85 years and found that the disease and congenital megacolon had no significant difference in clinical manifestations (9). The two patients diagnosed with ganglion cell deficiency in this study got their final pathological diagnose after surgery. In addition, it is reported that patients with ganglion cell deficiency is relatively older than other types, and female are more likely to get this disease (10). Etiologically, adult congenital megacolon is caused by the lack of ganglion cells in the distal colon, rectal submucosal nerve plexus, and myenteric nerve plexus (11). There are many types of adult congenital megacolon. Most patients are young and have received surgical intervention at their early ages (12). Only some short-segment and ultra-short-segment types with mild early symptoms don’t receive treatment in time and delay treatment to youth, which leads to the highly dilated normal intestinal segment at the proximal end of the diseased intestine and rubber-like intestinal wall due to compensatory thickening of the muscularis (13).

There are many similarities between adult idiopathic megacolon and adult congenital megacolon. In terms of symptoms, both have difficulty in defecation, abdominal pain, and bloating, which can be relieved after defecation (14–18). However, idiopathic megacolon does not have a significantly narrowed intestinal segment, and the expanded intestinal segment is the diseased one. The intestinal segment is characterized by a decrease in the number and degeneration of myenteric ganglia in the intestinal wall, normal acetylcholinesterase activity, thin smooth muscle layer of the intestinal wall, and weak bowel movements (19, 20). In the physical examination, the main difference lies in the disappearance of anorectal suppression reflex in adult congenital megacolon. It is important to follow the basic principles of surgery in the treatment of adult congenital megacolon. The purpose of surgery can be achieved by removing the stenotic, transitional, and obviously expanded segment.

According to our experience, the expanded and thickened intestinal segment needs to be completely removed to the normal intestinal segment at the proximal end. When tension exists at the anastomotic stoma, the splenic flexure of colon needs to be freed. In some cases, the proximal intestine should be removed to the transverse colon or even the ascending colon. According to recent reports, per-rectal endoscopic myotomy (PREM) is a safe and effective minimally invasive procedure with long-term response (21–23). The principle of surgical treatment of adult idiopathic megacolon is different from that of adult congenital megacolon. If the surgeons only remove the dilated intestine, the remained colon is likely to cause recurrence of the disease (24, 25). Subtotal colectomy with ileorectal anastomosis or ascending colorectal anastomosis is a better option. Considering the low anastomosis position in many patients with adult congenital megacolon, inadequate bowel preparation before surgery and long-term malnutrition, in our experiences, to reduce postoperative anastomosis related complications, it is recommended to perform a preventive terminal ileostomy during surgery. After the anastomosis is well-healed, the stoma will be returned.

The incidence of toxic megacolon is relatively low, and the causes include but not limit to ulcerative colitis, ischemic enteritis, collagenous colitis, and pseudomembranous enteritis caused by long-term use of antibiotics. However, the toxic megacolon progresses rapidly. Since the intestinal mucosal barrier is destroyed and bacterial toxins enter the blood, if the infection cannot be effectively controlled, it may lead to septic shock and even multiple organ failure (26). Among the three patients in this study, two of the mildly illed patients underwent colostomy, which was a damage-controlled stoma. Since a stoma could not effectively alleviate the infection in the other severely infected patient, subtotal colostomy was performed.

In obstruction caused by megacolon, some patients need emergency stoma surgery if conservative treatment is ineffective (27). In those included cases, 14 cases were admitted to hospital with emergency intestinal obstruction, accounting for 31.4% of patients with idiopathic megacolon and 37.5% of iatrogenic megacolon. Emergency operation is not suitable for each obstruction case, since the sever dilatation of the intestinal cavity is chronic and won’t perforate in a short period. Conservative treatments such as enema and colonoscopy decompression can be performed first, and enterostomy should only be selected when conservative treatment is ineffective.

It should be noted that some patients with idiopathic megacolon may be associated with malnutrition due to long-term constipation and fecal obstruction (28). For these patients, an intestinal stoma is a compelling choice. Although a stoma can temporarily relieve defecation problems, since diseased bowel is not removed during the enterostomy, the symptoms of abdominal distension and abdominal pain may still occur after the operation (29). In addition, because of the extreme expansion of the intestinal segment of the stoma, only a fenestration stoma can be performed. And after decompression, due to the retraction of the intestines and the tension of the abdominal wall, the stoma will retract and narrow. The fenestration stoma is acceptable as a temporary stoma for patients with planned second-stage surgery, but a loop stoma should be chosen as a permanent stoma.

Because the etiology of each patient is different, it is difficult for doctors to choose proper operative approaches and minimize the surgical risk. The choice of surgical methods is mainly depending on the patient’s condition, the basis of objective examination and the experience of the doctor team, only a reasonable surgical strategy can benefit the patient to the maximum extent.

## Data Availability Statement

The raw data supporting the conclusions of this article will be made available by the authors, without undue reservation.

## Ethics Statement

Written informed consent was obtained from the individual(s) for the publication of any potentially identifiable images or data included in this article.

## Author Contributions

WZ, JS, and SM contributed to conception and design of the study. SM and AP organized the database. YY and AP performed the statistical analysis. HG, LL, ZL, LH, RM, JS, and WZ provided patients’ data. All authors discussed the data. YY wrote the manuscript and subsequent revisions, which were reviewed by other authors. SM, YY, and AP contributed equally to this research. All authors read and approved the final manuscript.

## Conflict of Interest

The authors declare that the research was conducted in the absence of any commercial or financial relationships that could be construed as a potential conflict of interest.

## Publisher’s Note

All claims expressed in this article are solely those of the authors and do not necessarily represent those of their affiliated organizations, or those of the publisher, the editors and the reviewers. Any product that may be evaluated in this article, or claim that may be made by its manufacturer, is not guaranteed or endorsed by the publisher.
